# Supplementary material for: Household Cases Suggest That Cats Belonging to Owners with COVID-19 Have a Limited Role in Virus Transmission
Source: Viruses. 2021 Apr 14;13(4):673. doi: 10.3390/v13040673 (PMC8070925; doi:10.3390/v13040673)
Supplement: Supplementary file 1 [file viruses-13-00673-s001.zip › Instructions for owners.pdf]

CONSEILS POUR REALISER LES PRELEVEMENTS

☞ Utiliser des écouvillons stériles, secs, sans milieu de transport

Prélèvement oropharyngé :

1. Se désinfecter les mains avec la solution hydro alcoolique
2. **<u>Porter des gants</u>**
3. Retenez doucement le chat. En tenant la tête verticalement, abaissez la mâchoire inférieure pour ouvrir la bouche comme pour donner des médicaments sous forme de comprimés
4. À l'aide d'un **écouvillon stérile et sec**, insérez délicatement l'embout en profondeur dans **<u>l'oropharynx au niveau des amygdales et frotter fermement</u>**
5. L'écouvillon doit être visiblement humidifié, attestant du prélèvement de cellules épithéliales
6. Il faut veiller à éviter une forte contamination de salive par contact avec la langue
7. La plupart des animaux tolèrent bien cette procédure, bien qu'il faille veiller à éviter que le chat ne morde et n'avale des parties de l'écouvillon
8. Une fois prélevé, l'écouvillon est placé dans un **<u>contenant sec et stérile</u>**
9. Identifier le contenant en indiquant le nom du chat, la date de prélèvement **<u>et la zone prélevée</u>**
10. Conserver à +4°C avant envoi
11. Envoi sous couvert du froid (+ 4°C) dans les meilleurs délais ; envoi avec plaques de froid (surtout en été).

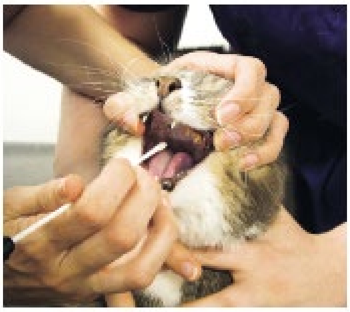

Prélèvement rectal :

1. Se désinfecter les mains avec la solution hydro alcoolique
2. **Porter des gants**
3. Pendant qu'un aide maintient le chat, le préleveur dégage la zone anale en maintenant doucement la queue
4. Enfoncer environ 1-2 cm **<u>l'écouvillon stérile et sec</u>** dans le rectum et faire un mouvement circulaire
5. Contrôler la présence de matière fécale sur le coton de l'écouvillon
6. Une fois prélevé, l'écouvillon est placé dans un **<u>contenant sec et stérile</u>**
7. Identifier le contenant en indiquant le nom du chat, la date de prélèvement **<u>et la zone prélevée</u>**
8. Conserver à +4°C avant envoi
9. Envoi sous couvert du froid (+ 4°C) dans les meilleurs délais ; envoi avec plaques de froid (surtout en été).
