# Supplementary material for: Household Cases Suggest That Cats Belonging to Owners with COVID-19 Have a Limited Role in Virus Transmission
Source: Viruses. 2021 Apr 14;13(4):673. doi: 10.3390/v13040673 (PMC8070925; doi:10.3390/v13040673)
Supplement: Supplementary file 1 [file viruses-13-00673-s001.zip › viruses-1150936-suppl.docx]

**Supplementary Table S1** Household descriptions.

| **Case** | **Numbers of Owners** | **Other Companions Animals** |
| --- | --- | --- |
| 1 | 2 | None |
| 2 | 1 | None |
| 3 | 1 | None |
| 4 | 1 | None |
| 5 | 2 | None |

**Supplementary Table S2.** Primers and probe used for RT-qPCR.

| **Gene** | **Primer Sequence** | **Reference** |
| --- | --- | --- |
| SARS-CoV-2 E | F: ACAGGTACGTTAATAGTT-AATAGCGT  P: FAM-ACACTAGCCATCCT-TACTGCGCTTCG-BBQ  R: ATATTGCAGCAGTACGC-ACACA | [23] |
| Cat RPS7 | F: GTCCCAGAAGCCGCACTT-TGAC  R: CTCTTGCCCACAATCTCG-CTCG | [24] |
| Human RPL30 | F: ACAGCATGCGGAAAATACTAC  R: AAAGGAAAATTTTGCAGGTTT | [25] |

RPL30: ribosomal protein L30; RPS7: 40S ribosomal protein S7. F: forward; R: reverse; P: probe.
